# Data‐driven regions of interest for longitudinal change in three variants of frontotemporal lobar degeneration

**DOI:** 10.1002/brb3.675

**Published:** 2017-03-23

**Authors:** Richard J. Binney, Aleksandr Pankov, Gabriel Marx, Xuanzie He, Faye McKenna, Adam M. Staffaroni, John Kornak, Suneth Attygalle, Adam L. Boxer, Norbert Schuff, Maria‐Luisa Gorno‐Tempini, Michael W. Weiner, Joel H. Kramer, Bruce L. Miller, Howard J. Rosen

**Affiliations:** ^1^ Department of NeurologyMemory and Aging CenterUniversity of California, San FranciscoSan FranciscoCAUSA; ^2^ Department of Epidemiology and BiostatisticsUniversity of California, San FranciscoSan FranciscoCAUSA; ^3^ Department of Neurological SurgeryUniversity of California, San FranciscoSan FranciscoCAUSA; ^4^ Department of RadiologyUniversity of California, San FranciscoSan FranciscoCAUSA

**Keywords:** frontotemporal dementia, longitudinal studies, magnetic resonance imaging, primary progressive aphasia

## Abstract

**Introduction:**

Longitudinal imaging of neurodegenerative disorders is a potentially powerful biomarker for use in clinical trials. In Alzheimer's disease, studies have demonstrated that empirically derived regions of interest (ROIs) can provide more reliable measurement of disease progression compared with anatomically defined ROIs.

**Methods:**

We set out to derive ROIs with optimal effect size for quantifying longitudinal change in a hypothetical clinical trial by comparing atrophy rates in 44 patients with behavioral variant of frontotemporal dementia (bvFTD), 30 with the semantic variant primary progressive aphasia (svPPA), and 26 with the nonfluent variant PPA (nfvPPA) to atrophy in 97 cognitively healthy controls.

**Results:**

The regions identified for each variant were generally what would be expected from prior studies of frontotemporal lobar degeneration (FTLD). Sample size estimates for detecting a 40% reduction in annual rate of ROI atrophy varied substantially across groups, being 103 per arm in bvFTD, 31 in nfvPPA, and 10 in svPPA, but in all groups were less than those estimated for a priori ROIs and clinical measures. The variability in location of peak regions of atrophy across individuals was highest in bvFTD and lowest in svPPA, likely relating to the differences in effect size.

**Conclusions:**

These findings suggest that, while cross‐validated maps of change can improve sensitivity to change in FTLD compared with a priori regions, the reliability of these maps differs considerably across syndromes. Future studies can utilize these maps to design clinical trials, and should try to identify factors accounting for the variability in patterns of atrophy across individuals, particularly those with bvFTD.

## 1 Introduction

Frontotemporal lobar degeneration (FTLD) is a neurodegenerative disorder that has a profound effect on the lives of patients and their families; one that can be considered more detrimental than the effects of more typical degenerative disease such as Alzheimer's disease (AD) because it is associated with an earlier age of onset (Papageorgiou, Kontaxis, Bonakis, Kalfakis, & Vassilopoulos, 2009) and more rapid rate of decline (Roberson et al., 2005). Neuroanatomically, it manifests distinctly from AD in that it primarily involves the frontal and anterior temporal cortex rather than medial temporal and temporoparietal regions. There are no approved treatments for FTLD but efforts to develop them are underway (Boxer & Boeve, 2007; Boxer, Gold, et al., 2013; Boxer, Knopman, et al., 2013).

Brain imaging is a powerful tool in neurodegenerative disease. MRI and PET, the most commonly used techniques, can be used to support diagnosis, and measures derived from brain images correlate with the type and severity of symptoms in each patient (Tartaglia, Rosen, & Miller, 2011). These observations have led to studies examining the utility of longitudinal brain imaging as an outcome measure for clinical drug trials, which have demonstrated that MRI can track change in neurodegenerative disorders more reliably than clinical measures such as cognitive testing (Knopman et al., 2009; Weiner et al., 2013).

One limitation of brain imaging is that each image produces hundreds or thousands of data points per patient corresponding to spatial locations in the brain, posing a significant hurdle for defining imaging‐based biomarkers (Friston, Holmes, Poline, Price, & Frith, 1996). One of the most common approaches to reduce the large‐scale data in imaging studies is to limit measures of change to aggregated estimates over regions of interest (ROIs), which tend to be chosen based on prior knowledge about the regions that are most severely affected in each disease. In AD, ROIs chosen often include the hippocampus, entorhinal cortex, and temporoparietal regions (Dickerson et al., 2011). In FTLD, the frontal and/or temporal lobes have been used (Gordon et al., 2010; Krueger et al., 2010). However, the regions most severely affected in each disease tend to be those affected earliest (Jack et al., 1997; Seeley et al., 2008). When a disorder moves beyond the earliest stages, it is possible that regions affected early begin to slow their rate of change while other regions, previously only mildly affected, begin to accelerate their decline (Brambati et al., 2009; Rohrer et al., 2012; Schuff et al., 2012). Thus, ROIs chosen based on regions that are most strongly associated with the disease may not be optimal for determining treatment effects. Recent studies have shown that empirically derived ROIs representing the most reliable voxels associated with an effect of interest can be used to improve diagnosis of dementia (Avants, Cook, Ungar, Gee, & Grossman, 2010; McMillan et al., 2014) and to improve statistical power for longitudinal analysis (Chen et al., 2010; Hua et al., 2009) compared with ROIs chosen based on their general association with the disease. We recently created an empirically based ROI of annualized atrophy in a group of FTLD patients and demonstrated the potential for larger effect sizes than a priori ROIs (Pankov et al., 2016).

Frontotemporal lobar degeneration includes a spectrum of disorders with varying molecular, clinical and imaging characteristics (Bang, Spina, & Miller, 2015; Tartaglia et al., 2011). The three canonical clinical presentations include: (1) the behavioral variant of frontotemporal dementia (bvFTD), characterized by progressive impairment in socioemotional function; (2) the semantic variant of primary progressive aphasia (svPPA; also known as semantic dementia), characterized by progressive loss of knowledge about words and objects, and (3) the nonfluent variant of PPA (nfvPPA), characterized by progressive impairment of articulation and speech (Gorno‐Tempini et al., 2011; Rascovsky et al., 2011). Each variant is associated with distinct distributions of cortical atrophy varying particularly in the degree of temporal and frontal lobe involvement. BvFTD alone can show highly variable patterns of atrophy (Whitwell et al., 2011). Therefore, it is likely that the most sensitive ROIs for FTLD will be derived empirically from and specific to each variant. In our previous analysis (Pankov et al., 2016), we examined annual volume loss in a mixed group of bvFTD and svPPA cases. The number of subjects in that study was too small to examine syndrome‐specific patterns of change. In this study, we set out to identify the most reliable regions of change separately in bvFTD, svPPA, and nfvPPA and estimate sample sizes for theoretical clinical trials that might involve each of these groups individually.

## 2 Methods

### 2.1 Subjects

Subjects in this retrospective study included any subject studied at the UCSF Memory and Aging Center (MAC) who had undergone MRI twice over a period ranging between 6 months and 2 years with a diagnosis of behavioral variant of bvFTD (*n* = 44), svPPA (*n* = 30), or nfvPPA (*n* = 26). All data were annualized prior to analysis. In addition, we assembled a group of healthy comparison subjects (HC) with longitudinal imaging with the same age range and sex distribution of the FTLD group (HC, *n* = 97, mean age 64.77 ± 6/95, mean education level 17.65 ± 6.95). Patients included in this study were recruited between 2008 and 2015 through ongoing studies (AG019724, AG032306, AG023501) at the MAC. Diagnosis for these studies was based on a multidisciplinary evaluation incorporating neurological, neuropsychological, and nursing assessment (Rosen et al., 2002). Structural brain imaging was not used to make syndromic diagnosis, but only to exclude other causes of brain damage, such as strokes or tumors. Disease duration was estimated based on the year of initial symptoms provided by the patient or their informant. HC data were obtained from a cohort of subjects recruited at the MAC via advertisements and community events. HCs underwent the same evaluation as patients and were required to have no clinically significant cognitive or behavioral complaints, performance within one standard deviation of normal on all cognitive tasks, and to have brought a knowledgeable informant to verify the absence of clinically significant cognitive or behavioral problems. HCs were excluded if they had a history of significant mood disorders, clinically significant alcohol or drug use, significant vascular disease, visual problems that would impair test performance, other neurologic conditions, and self‐reported deficits in cognition.

All subjects were required to have had two T1‐weighted MRI scans acquired with the same scanner and pulse sequence and with a quality suitable for processing. Images were inspected for quality, including ensuring whole‐brain coverage and looking for excessive motion artifact. Assessment of CNS amyloid burden, usually with PET amyloid imaging using Pittsburgh B compound was available in 63 of the patients. Because the goal of the analysis was to examine the change maps in groups with specific clinical diagnoses, all patients with available MRI data were included, regardless of amyloid status. A sensitivity analysis was conducted on the subset of bvFTD patients who were known to be amyloid negative to examine whether maps of change differed substantially from the maps created from the group as a whole. Amyloid status was generally not available in the controls. All research was performed in accordance with the Code of Ethics of the World Medical Association. All subjects provided informed consent, and the clinical and imaging protocols were approved by the UCSF Committee on Human Research.

### 2.2 Clinical assessment

Patients were diagnosed using published criteria (McKhann et al., 1984; Neary et al., 1998) after a comprehensive evaluation at the UCSF MAC including neurological history and examination, nursing assessment, laboratory evaluation, and a previously described neuropsychological assessment (Kramer et al., 2003). The neuropsychological assessment battery includes the Mini Mental State Examination (MMSE) (Folstein, Folstein, & McHugh, 1975), and tests tapping into functions relevant to FTLD including memory, language and frontal/executive functions. These include list‐learning (California Verbal Learning Task [CVLT]; Delis, Kramer, Kaplan, & Ober, 2000), confrontational naming (15 items from the Boston Naming Test [BNT]; Kaplan, Goodglass, & Wintraub, 1983), set‐shifting (modified version of the Trails B task; Kramer et al., 2003), and tests of lexical fluency (words beginning with the letter “D”; Birn et al., 2010), and semantic fluency (animals; Delis, Kaplan, & Kramer, 2001). Functional state was quantified using the Clinical Dementia Rating (CDR; Morris, 1997), which was used here to generate a continuous variable based on the sum of the individual ratings for functional domains, typically referred to as the sum‐of‐boxes (CDR‐SB). Although an FTLD‐specific version of the CDR has been developed (Knopman et al., 2008), many of these patients were assessed before our center began using it, so this analysis was done using only the traditional CDR domains.

### 2.3 Image acquisition

A 3.0T MRI was acquired on a Siemens Tim Trio system (Siemens, Iselin, NJ, USA) equipped with a 12‐channel receiver head coil. A volumetric MPRAGE sequence was used to acquire T1‐weighted images of the entire brain (coronal slice orientation; slice thickness = 1.0 mm; in‐plane resolution = 1.0 × 1.0 mm; matrix = 240 × 256; TR = 2,300 ms; TE = 3 ms; TI = 900 ms; flip angle = 9°).

### 2.4 Image processing

Longitudinal changes in regional brain volume were estimated using the Pairwise Longitudinal Registration Toolbox implemented in SPM12 (Ashburner & Ridgway, 2012), which addresses concerns regarding asymmetric bias in pair‐wise longitudinal registration (Thomas, 2010; Yushkevich et al., 2010). The process begins with intrasubject registration using iterative and interleaved rigid‐body alignment, diffeomorphic warping, and correction for differential intensity inhomogeneity to generate a within‐subject template representing an average of the subject's two scans with respect to position, shape, and intensity nonuniformity. Two Jacobian determinant maps are then computed; one that encodes the relative difference in volume between the first scan and the within‐subject average, and another that describes the relative volume between the second scan and the average. Computing the difference between these two Jacobian determinants provides a map of relative change in volume between scan one and scan two at each spatial location. The change maps were divided by the interscan interval (in units of years) to become maps of annual rate of relative volume change. Each subject's average image was bias‐corrected and the brain was partitioned into gray matter, white matter, and cerebrospinal fluid (CSF), using SPM12's unified segmentation procedure. The contraction/expansion maps were then multiplied with the gray matter probabilistic tissue segmented maps on a voxel‐by‐voxel basis, in within‐subject average space, to restrict analyses to cortical and subcortical gray matter.

Image segmentation can be affected by several factors that may relate to disease, including histological abnormalities that could cause changes in tissue contrast, as well as subject movement, which would decrease signal‐to‐noise ratios. To ensure that the analysis would not be excessively influenced by differences in the quality of gray matter segmentations across groups, we reviewed the distributions of values for the whole‐brain gray matter probability maps across groups. The shapes of these distributions were similar across groups.

To allow statistical analysis across subjects, all images were transformed to a standardized space. Mappings from the gray matter and white matter segments of the within‐subject averages (all patients and control subjects) to an iteratively evolving study‐specific population mean of these tissues were estimated using the DARTEL (diffeomorphic anatomical registration through an exponentiated lie algebra) toolbox (Ashburner, 2007). DARTEL minimizes the geodesic distance from each patient to the population mean. Thus, between‐population asymmetries in registration, which could also lead to erroneous population effects, were addressed. An affine mapping between the population mean and MNI space (defined by SPM12's Prior Tissue Probability Map) was also estimated and combined with each subject‐to‐population mean mapping for warping average images and volume expansion/contraction rate maps to MNI space. The rate change maps were then warped to population‐in‐MNI space using the abovementioned mapping composition, and resampled to 1.5 mm^3^ without “volume‐preserving” modulation. No spatial smoothing was applied. Subsequent analysis was done using only the gray matter maps of each patient.

### 2.5 Generation and evaluation of a data‐driven ROI

#### 2.5.1 Overview

Our data‐driven ROI generation procedure follows (in spirit) from prior approaches where optimal effect sizes were estimated from a training set and tested on an independent test set (Chen et al., 2010; Hua et al., 2009). However, we use a cross‐validation‐type scheme rather than a simple training‐test approach in order to maximally use the data available in generating a “best” consensus ROI; we thereby avoid overfitting for our estimates of effect size and sample size. For each randomly partitioned cross‐validation training set, we first generated a Student's *t* statistic (allowing unequal group variances) at each voxel in standardized space. The map of *t* statistics quantifies the difference in the effect size of contraction between each FTLD patient group and HCs across the brain. A 3D ROI is extracted by thresholding the map of *t* statistics such that the threshold used maximizes the effect size in the same training set. The effect size for tissue contraction over 1 year is then estimated on the independent test set partition of the data. After repeating the process multiple times, the effect size is estimated as the mean of the estimates across the independent test sets. A consensus‐weighted ROI was then generated from the cross‐validation procedure by weighting each voxel based on its reliability in distinguishing contraction between patient and HC groups across the random partitions.

We specifically chose to examine only contracting voxels because expanding voxels would often represent residual CSF spaces that were not completely removed by segmentation and masking. If we included expanding voxels, we would be making the assumption that future studies would encounter similar patterns of expansion in residual/unmasked CSF voxels. Thus, the generalizability of the resultant map would be dependent on similarity between our segmentation and masking procedures and the segmentation outcomes of future studies. Given that this segmentation accuracy would depend on many factors, we felt that limiting the ROI to only voxels that would be expected to contract would be more conservative and generalizable.

#### 2.5.2 Procedure

**Figure 1:**
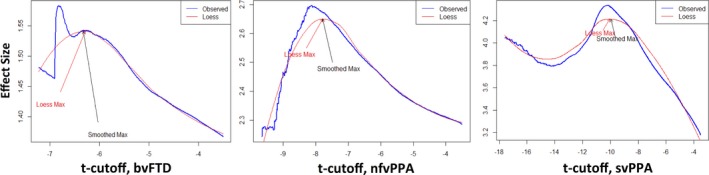
Plots of effect size versus *t* score threshold cutoff for each clinical variant, used to identify *t* score threshold giving map with maximum effect size

Data‐driven ROIs were generated separately for each clinical variant of FTLD by comparing change maps in each patient group to change in the entire control group. The cross‐validation algorithm proceeded as follows:

1. For each patient group, the combined set of control and patient data were randomly divided into training and test sets, with 16% of the data being assigned to the test set. Each split was stratified such that the proportion of FTLD to normal samples was required to be more than 1/3, but less than 2/3 of the total test set. For example, in the case of bvFTD where we have the *N* of 97 for controls and 44 for bvFTD, the size of the test set would be (97 + 44) × 0.16 = 23 images, of which 1/3 (8) to 2/3 (15) would have to be bvFTD.
2. A series of ROIs was then generated in each training set by thresholding the *t*‐maps over a set of levels ranging from 3.5 to the maximum observed *t* statistic in increments of 0.01 units.
3. The effect size for the mean difference in rate of change between each FTLD variant and controls was then calculated for each ROI of the training set using Cohen's *d*. A plot is then generated of effect size versus each *t* statistic cutoff. The plot represents the relationship between the *t* statistic cutoff and the corresponding effect size for each resulting ROI (see below, Figure 1).
4. The ROI associated with the *t* statistic cutoff corresponding to the maximum effect size is selected.
5. The ROI from step 4 is then used to calculate the effect size in the test set to obtain an unbiased effect size estimate for the particular partition.

Steps 1–5 were then repeated 1,024 times, reassigning patients into the training and test sets each time. At the end of the process, we have a set of “optimal” ROIs (across training/test set partitions). The effect size is then estimated as the mean effect size over all partitions. To then estimate a consensus ROI from the ensemble of cross‐validated measurements, we weighted the contribution of each voxel to the data‐driven ROI as the proportion of cross‐validation partitions (weighted by the effect size for that cross‐validation sample) in which the voxel contributes to the consensus ROI. Thus, the resulting map has a stronger representation from voxels consistently contributing to the overall effect size across cross‐validation samples and weaker representation from voxels whose contribution was more variable.

It should be noted that at high *t*‐thresholds the maximal empirical effect size estimate becomes highly variable over neighboring thresholds because only a small number of voxels form a ROI at high thresholds. To mitigate this effect and generate a stable estimate of maximum effect size, we smoothed the effect size curve plotted against threshold. However, even lowess regression did not sufficiently downweight the influence of high thresholds. We therefore implemented a heuristic method to identify the maximum effect size. Specifically, a lowess regression was performed after iteratively excluding a top set of voxels (from 0% to 10% of the highest voxels in increments corresponding to those associated with the *t*‐thresholds). At each iteration, the lowess‐smoothed maximum was calculated, and the overall maximum was taken as the median of all the smoothed maximums. This approach was able to identify the location of the maximum in reasonable agreement with the choice that one would make visually as being the maximum of the relatively smooth (and therefore reliable) part of the curve (see Figure 1).

In order to estimate the potential impact of using an optimized data‐driven ROI of change for future clinical trials, we calculated the necessary sample size in a hypothetical clinical trial seeking to detect a 20% and 40% reduction in the change over 1 year in volume loss in each FTLD group (α = 0.05, power = 0.8). We compared the sample size from the effect size estimated using the data‐driven ROIs (i.e., via the mean effect size over the test set estimates) to the sample sizes obtained by measuring change within a priori ROIs based on cerebral anatomy. For this purpose, we used frontal, temporal, combined frontal and temporal, and whole gray matter masks as regions of interest (ROIs) relevant to FTLD. These ROIs were obtained from the AAL brain atlas supplied with the WFU‐PickAtlas software package (Maldjian, Laurienti, Kraft, & Burdette, 2003).

### 2.6 Change in clinical variables and sample size estimates

Changes in clinical variables were analyzed using linear mixed effects models with cognitive score as the dependent variable and elapsed time in years as the predictor. In order to compare the sample size estimates generated for imaging‐based measures of change to those generated using clinical measures, we calculated sample size estimates using annualized changes in score for the MMSE, selected measures of language and executive functioning, and for the CDR, which has been identified as an attractive measure for tracking change in FTLD (Knopman et al., 2008). We calculated the necessary sample size in a hypothetical clinical trial seeking to detect a 20% and 40% reduction in the change over 1 year in clinical measures in each FTLD group (α = 0.05, β = 0.8). These analyses were carried out using Stata (version 14, www.stata.com).

## 3 Results

### 3.1 Group demographics and clinical assessments

Demographic characteristics and cognitive testing performance in the patient groups are presented in Table 1. The mean age for the control group was 64.4 (±7). The bvFTD group was slightly younger than the controls (−3.69 years, 95% CI [−6.23, −1.14], *p* = .005) and the nfvPPA group was slightly older (+3.73 years, 95% CI [0.66, 6.8], *p* = .018). The differences in mean interscan interval across groups were not statistically significant (*p* = .11), nor were differences in education level (*p* = .43) or disease duration (*p* = .45). In terms of cognitive and functional data, scores were generally what would be expected. SvPPA patients tended to score more poorly on measures of verbal episodic memory and language compared with the other groups. BvFTD and nfvPPA patients performed more poorly on measures of executive function than svPPA. BvFTD showed the most functional impairment, as measured by the CDR‐SB. Annualized changes over time were significant in bvFTD for MMSE (−3.25, 95% CI [−5.26, −1.24], *p* = .001), CDR (1.73, 95% CI 0.95, 2.5], *p* < .001), CVLT‐long delay (−0.82, 95% CI −1.35, −0.3], *p* = .002), and semantic fluency (−1.61, 95% CI −2.13, −0.08], *p* = .039). In svPPA, changes were significant for MMSE (−4.40, 95% CI [−5.85, −2.95], *p* < .001), CDR (1.32, 95% CI [−0.45, 2.19, *p* = .003), BNT (−1.36, 95% CI [−2.05, −0.67], *p* < .001), and semantic fluency (−1.62, 95% CI [−2.31, −0.94], *p* < .001). In nfvPPA, changes were significant for CDR (1.61, 95% CI [0.7, 2.52], *p* = .001), BNT (−1.66, 95% CI [−3.17, −0.16], *p* = .03), semantic fluency (−1.83, 95% CI [−3.35, −0.33], *p* = .017), and lexical fluency (−1.05, 95% CI [−1.19, −0.19], *p* = .17).

**Table 1:** Baseline and 1‐year clinical data in patient groups

	bvFTD (*n* = 44)		nfvPPA (*n* = 26)		svPPA (*n* = 30)	
Demographics						
Age at year 1 (*SD*)	61.14 (7.36)		71.6 (7.73)		66.7 (6.69)	
Sex (M/F)	25/19		12/14		16/14	
Education	15.88 (2.95)		16.58 (2.76)		16.76 (3.21)	
Disease duration	5.66 (3.85)		4.81 (2.87)		6.05 (4.2)	
Mean interscan interval	1.09 (0.31)		1.14 (0.38)		1.07 (0.38)	

Measure	Baseline	Follow‐up	Baseline	Follow‐up	Baseline	Follow‐up
Cognitive testing						
MMSE	24.57 (4.19)	21.94 (7.09)^b^	25.32 (4.66)	23.05 (6.85)	25.03 (3.89)	19.96 (7.33)^b^
CDR‐SB	6.67 (2.9)	8.59 (3.22)^b^	2.38 (2.14)	4.02 (3.7)^b^	4.11 (2.28)	5.72 (3.15)^b^
CVLT‐LD^a^ (max = 9)	4.02 (2.8)	3.55 (3.05)^b^	5.48 (2.92)	5.19 (2.91)	1.38 (2.0)	1.21 (2.25)
BNT (max = 15)	12.11 (3.43)	11.64 (4.49)	12.08 (2.84)	10.48 (4.32)^b^	4.54 (3.36)	3.11 (3.36)^b^
Semantic fluency	10.45 (6.26)	9.66 (7.0)^b^	10.33 (7.25)	9 (7.05)	6.85 (4.57)	5.5 (5.56)^b^
Lexical fluency	7 (4.48)	6.56 (5.56)	6.16 (4.7)	5.27 (5.13)^b^	7.77 (4.06)	6.73 (4.97)
Trails set‐shifting	70.9 (40.43)	76.53 (42.55)	71.88 (35.3)	64.38 (33.93)	49.08 (29.13)	50.01 (29.76)

bvFTD, behavioral variant of frontotemporal dementia; nfvPPA, nonfluent variant of primary progressive aphasia; svPPA, semantic variant of primary progressive aphasia; BNT, Boston Naming Test; CVLT, California Verbal Learning Task; CDR, Clinical Dementia Rating.

a LD = long delay (10 min).

b *p* < .05 for change between baseline and follow‐up.

### 3.2 Change maps and effect sizes

As expected, there was a clear relationship between the *t* statistic threshold and the effect size for the associated ROI in each FTLD variant. For illustrative purposes, Figure 1 depicts this relationship in each variant developed from the complete dataset (step 2 of the algorithm; no training/test partitioning has been performed in generating the plot. Rather, the full dataset was used in order to maximally extract information from the data).

**Figure 2:**
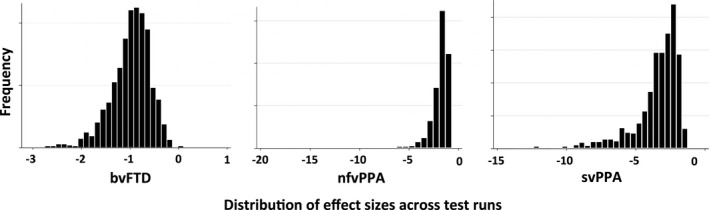
Histograms of effect size calculations across cross‐validation runs in each diagnostic group

Figure 2 depicts the range of effect sizes obtained by applying the ROIs obtained from training sets to test sets (step 5 of the algorithm) over 1,024 iterations. The mean effect sizes were −0.98 (95% CI [−0.96, −0.98]) for bvFTD, −1.84 (95% CI [−1.78, −1.9]) for nfvPPA, and −3.45 (95% CI [−3.35, −3.55]) for svPPA.

**Figure 3:**
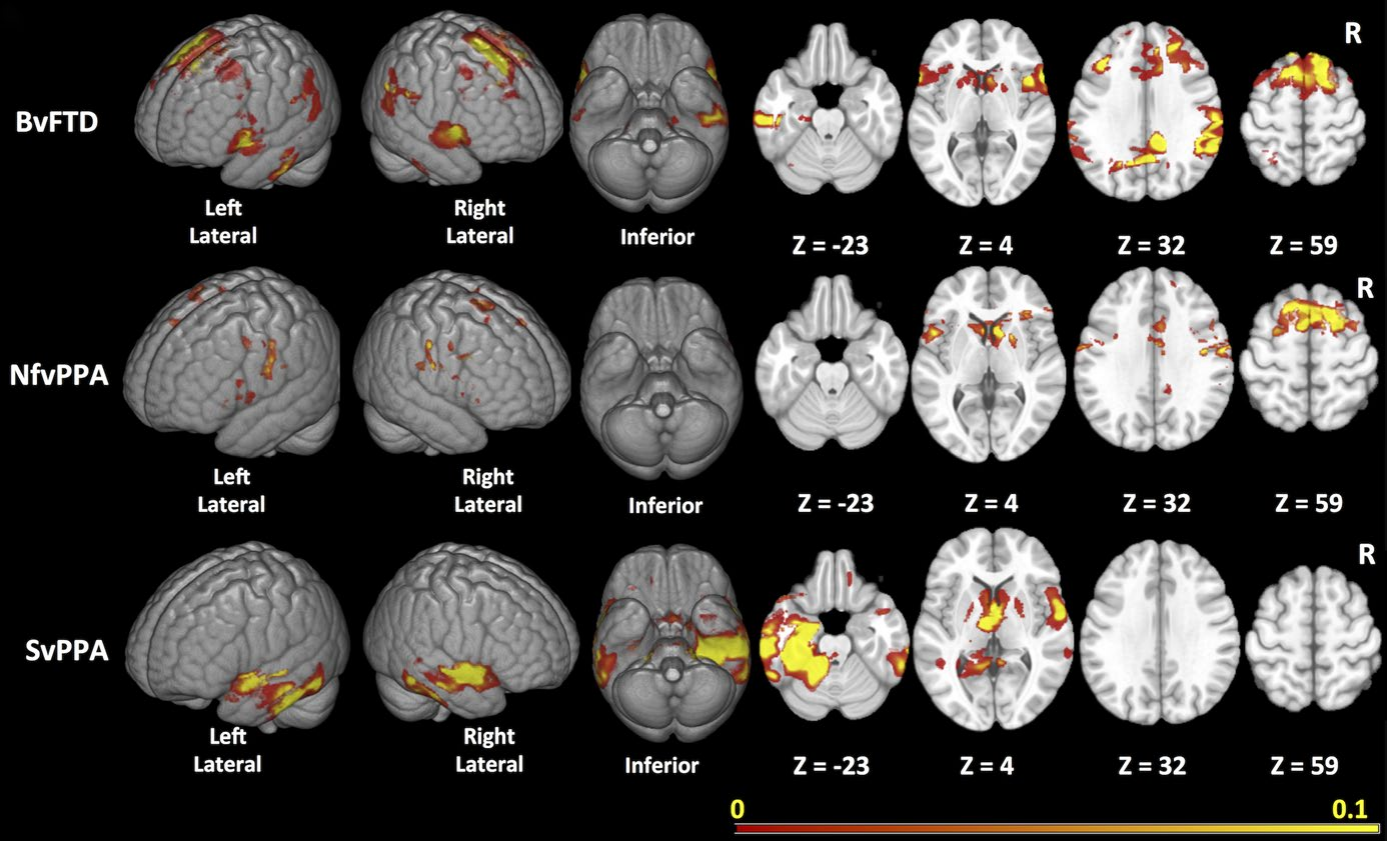
Maps of consensus regions of interest for the three main variants

Figure 3 depicts the consensus ROIs created for each variant. The ROI maps are displayed on a scale representing the weighing for each voxel. In bvFTD, the regions in the optimal ROI included medial and lateral portions of the frontal cortex, the perisylvian regions including the insula, and the striatum, in particular the caudate heads, with no inclusion of the orbitofrontal surface. In addition, the map included portions of the temporoparietal junctions, medial parietal cortex, and mid‐inferolateral temporal region. In nfvPPA, the most reliable regions of change were identified in the dorsal portions of the medial wall of the frontal lobes, and on the lateral frontal lobes primarily in the precentral regions with extension into the perisylvian region, and also caudate head involvement. In svPPA, the optimal ROI included superior and ventral anterior temporal cortex (but only partially included the temporal polar cortex) and mid‐to‐posterior inferolateral portions of the temporal lobes. The ROI in svPPA was bilateral but more extensive on the left. It also extended into the ventromedial frontal and caudate regions.

Table 2 compares the sample size estimates for a hypothetical 1‐year 1:1 parallel group trial designed to detect a 20% or 40% reduction in rate of decline obtained using the statistical ROIs (taken as the mean of the test effect sizes from the cross‐validation procedure) with those obtained using anatomically based ROIs and clinical data. In every variant, the sample size estimated with the statistically derived ROI was lower than sample sizes from frontal and/or temporal, or whole gray matter ROIs. The improvements were larger for nfvPPA and svPPA compared with bvFTD. For instance, in nfvPPA, the data‐driven ROI resulted in a 31% reduction in the sample size required to see a 20% reduction in atrophy in a theoretical clinical trial when compared with the best a priori ROI (118 patients per arm vs. 170 using temporal gray matter). In svPPA, the sample size needed was reduced by 53% (34 patients per arm vs. 73 using temporal gray matter). In bvFTD, the data‐driven ROI improved the sample size estimate by 21% (409 patients per arm vs. 521 using whole gray matter). These results can be compared with the sample size estimates required to achieve a 20% or 40% reduction in rate of decline for MMSE, CDR‐SB, and other cognitive tasks (Table 2). The sample sizes are substantially larger than those required for imaging.

**Table 2:** Sample size calculations^a^ (per arm) for rate of atrophy in a priori and data‐driven regions of interest, and for selected clinical measures^b^

	bvFTD		nfvPPA		svPPA	
	Sample size 20% reduction	Sample size 40% reduction	Sample size 20% reduction	Sample size 40% reduction	Sample size 20% reduction	Sample size 40% reduction
Imaging measures						
Frontal lobe	593	149	191	49	346	88
Temporal lobe	564	142	170	44	73	19
Frontal/temporal	755	190	507	128	94	25
Whole gray	521	131	172	44	111	29
Data‐driven	**409**	**103**	**118**	**31**	**34**	**10**
Clinical measures						
MMSE	2,090	523	3,457	893	546	137
CDR‐SB	**592**	**152**	**1,522**	**367**	776	194
BNT	3,340	835	4,728	1,182	841	211
Category fluency	1,795	449	1,572	393	**426**	**107**
Phonemic fluency	3,650	913	3,290	823	2,256	564
Modified trails time	2,132	533	863,592	215,898	1,169	293

bvFTD, behavioral variant of frontotemporal dementia; nfvPPA, nonfluent variant of primary progressive aphasia; svPPA, semantic variant of primary progressive aphasia; BNT, Boston Naming Test; CDR, Clinical Dementia Rating.

a Sample size for placebo‐controlled trial with 1:1 treated/placebo ratio, standard deviation based on patient group only (see Section 2).

b The imaging measure with the highest effect size for each diagnostic group is highlighted (bold) to facilitate comparison.

### 3.3 Change maps in amyloid‐negative and nongene carrier bvFTD subjects

**Figure 4:**
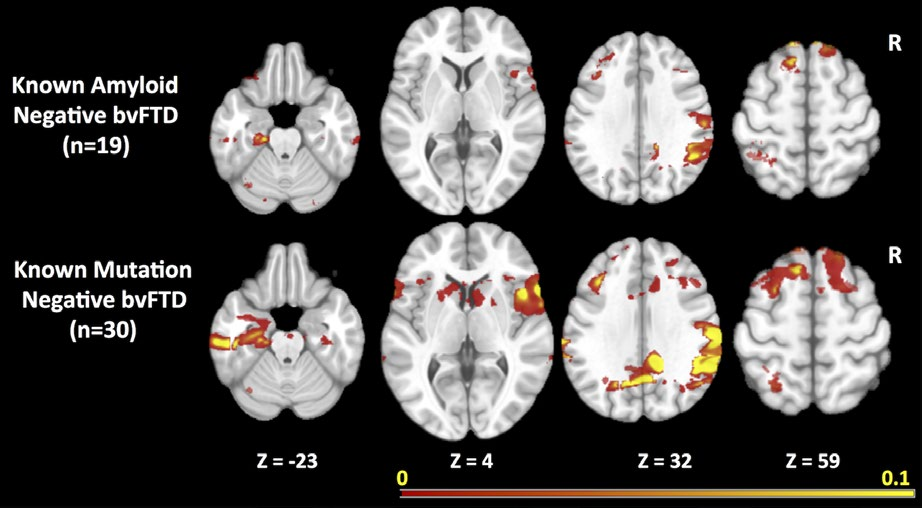
Maps of consensus regions of interest for behavioral variant of frontotemporal dementia sensitivity analysis using amyloid‐negative and gene‐negative subgroups

The prominence of longitudinal atrophy in the parietal regions in bvFTD raised concerns that the change maps might be influenced by individuals diagnosed with bvFTD who were amyloid positive. Of the 44 bvFTD patients, 20 had amyloid imaging and one of them was amyloid positive. We performed a sensitivity analysis examining rates of change in the 19 known amyloid‐negative bvFTD patients versus controls. Although the resulting change map included fewer voxels, likely due to a relatively small number of subjects being used for the cross‐validation, the resulting change map (Figure 4, top row) included a similar set of regions as the overall bvFTD map, including temporal regions and lateral and medial parietal regions.

Similarly, previous reports have demonstrated that patterns of atrophy in autosomal dominant forms are different than in sporadic FTD, with more widespread cortical involvement, including the parietal lobes. All of the bvFTD cases had genetic testing performed through research using previously described methods (Naasan et al., 2016). Fourteen of the 44 bvFTD patients were gene carriers. To examine whether mutation carriers were having a strong effect of increasing the likelihood of parietal changes, we performed another sensitivity analysis of the group of 30 bvFTD subjects after removing individuals with mutations. Again, the resulting change map (Figure 4, bottom row) was similar to the map for the bvFTD group as a whole.

### 3.4 Variability in locations of peak change across individuals

**Figure 5:**
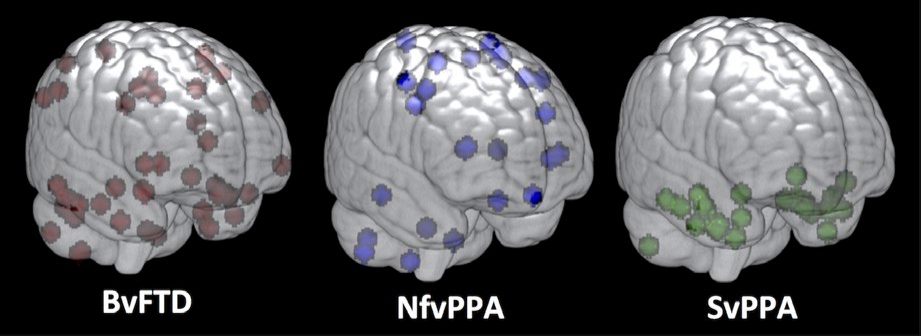
Maps of peak regions of longitudinal atrophy across patients in each of the three major variants

The variability in effect size across clinical syndromes was striking. One possible explanation is that mean rates of change were slower for bvFTD than for other groups; however, this would be inconsistent with prior studies indicating that rates of decline in clinical measures and brain volume in bvFTD are similar to rates of decline in other variants (Krueger et al., 2010; Rascovsky et al., 2001; Roberson et al., 2005). Given that the algorithm is designed to quantify the reliability of change in each voxel across individuals, another possibility is that the patterns of change might vary across individuals differently in each of the groups. To examine this, we plotted the locations of peak voxels (i.e., those with the highest rate of change) for all individuals, and displayed these locations in MNI space for each diagnostic group (Figure 5). As would be predicted from the effect size estimates, peak regions of change were highly clustered across individuals in the svPPA group, but with greater spatial variation in the locations of peaks in nfvPPA, and perhaps the most heterogeneous spatial distribution was seen in bvFTD.

## 4 Discussion

The aim of this analysis was to create ROIs that would generate maximal effect sizes for measuring change in cortical volume in three major variants of FTLD. As would be expected, the maps varied considerably across the three major variants. In bvFTD, they included the medial and lateral portions of the frontal lobes, the insula, the striatum, and the temporoparietal regions bilaterally. In svPPA, the most reliable change occurred primarily in ventral and lateral temporal, and medial frontal regions, and in nfvPPA, the changes occurred in the medial and lateral portions of the frontal lobes with predominant involvement of the precentral and perisylvian regions. Estimated effect sizes within these optimal ROIs varied considerably, being highest in svPPA and lowest in bvFTD. The main factor likely contributing to the differences in effect size appeared to be the level of spatial variability in atrophy locations across individuals, with bvFTD and nfvPPA showing the most widely distributed patterns of change. In all analyses, the sample size estimates for a theoretical clinical trial obtained using the statistical ROI approach were smaller than the estimates obtained with a priori lobar ROIs.

The specific regions identified in the change maps were generally what would be expected from prior cross‐sectional and longitudinal studies of FTLD, with some notable exceptions. In bvFTD, there was surprisingly little involvement of the orbitofrontal regions, while there was change detectable in the posterior temporoparietal regions. Current models propose that the pattern of change in structural MRI in neurodegenerative diseases follows a nonlinear pattern, with acceleration of change somewhere near the point of symptom onset, and deceleration of change in the later phases of illness (Jack et al., 2013). Given that atrophy in bvFTD occurs earliest in the insula and ventromedial frontal regions (Kril & Halliday, 2004; Seeley et al., 2008), these regions may reach a point where additional volume loss does not occur, while at the same time regions that are not involved early in bvFTD, such as the parietal lobes, may just be entering the phase of rapid decline when patients typically present for evaluation. The same phenomenon may explain the relative sparing of the temporal poles in the change maps for svPPA, which has been observed in prior studies and attributed to floor effects (Brambati et al., 2009; Rohrer et al., 2008). These findings highlight the value of empirically defined ROIs in tracking change as opposed to using ROIs defined according to prior knowledge about the regions that are most severely affected in each disease. These ROIs are affected by regional patterns of acceleration and deceleration that are likely stage specific, and thus would need to be recreated for use in patient groups substantially earlier or later in the disease course than those studied here.

Perhaps, more striking than the regions identified were the differences in sample size estimates across syndromes. Our data indicate that the sample sizes that would be required to detect changes in the rate of atrophy in bvFTD are larger than in nfvPPA and even more so when compared with svPPA. The fact that estimates obtained using the statistically driven approach were only slightly better than those obtained with whole gray matter supports the idea that the variability in regions of change in bvFTD makes it difficult to find focal, reliable regions for bvFTD as a whole. In contrast, in nfvPPA and particularly svPPA, the stronger overlap in regions of peak atrophy between individuals means that very reliable change can be measured in a relatively circumscribed region, such that techniques designed to find these regions, like the one used in this analysis, yield significant benefits for clinical trials.

The reason for the low level of predictability in regions of change across individuals with bvFTD is not readily apparent. Based on our analysis, the presence of amyloid‐positive cases or mutation carriers were not likely explanations because the maps generated using only known amyloid‐negative and known gene‐negative cases were similar to those obtained in bvFTD as a whole, including the presence of atrophy in the parietal lobes. Of course, we may still have included some cases due to mutations not yet discovered. Variability in the causative proteinopathy across individuals may be another explanation. Although svPPA is almost uniformly associated with Tar‐DNA‐binding protein type C (TDP‐C) protein pathology, bvFTD can be associated with a variety of proteinopathies including various forms of TDP as well as various forms of tau pathology including progressive supranuclear palsy, corticobasal degeneration, Pick's disease, and other variants (Bang et al., 2015). Differences between proteinopathies in patterns of imaging abnormalities have been established cross‐sectionally (Whitwell et al., 2011). Patterns of decline across different proteinopathies can also be examined as cohorts of autopsied cases with longitudinal imaging data grow, and techniques for identifying specific proteinopathies in vivo improve. In addition, current theories suggest that proteins causing neurodegenerative disease spread within neuroanatomically defined networks (Seeley, Crawford, Zhou, Miller, & Greicius, 2009). It is possible that the particular network involved in a disorder, and/or variability in strengths of connectivity within and between networks across individuals may also mediate patterns of spread. Verification that any of these, or other factors, can predict individual patterns of change would have obvious benefit for future clinical trials. It is also possible that other imaging methods, such as diffusion tensor imaging, may provide more reliable methods of tracking change over time (Mahoney et al., 2015).

One potential benefit from the use of imaging as a marker of longitudinal decline is that increased precision could result in improved effect sizes when compared with clinical measures of change (Weiner et al., 2013). This was generally confirmed in our analysis. For instance, we found that a placebo‐controlled trial would require 592 subjects per arm using the CDR‐SB to detect a 20% reduction in rate of change in bvFTD (Table 2). This estimate is roughly consistent with a prior study that estimated a sample size of 582 (Gordon et al., 2010) to detect a 25% effect of a drug. In contrast, our analysis indicates that a study measuring rates of atrophy using a statistically derived ROI in T1‐weighted images would require 409 people to detect the same effect. That said, other groups have published methods for identifying optimal clinical measures for tracking change using methods that are similar in principle to the approach used here for brain voxels (Ard, Raghavan, & Edland, 2015). These have yet to be examined in FTLD. While it is currently unlikely that volumetric change would be acceptable as a primary endpoint in clinical trials, this might become possible if reliable links between volumetric changes and clinical changes can be established. In addition, imaging could be used as evidence for a disease modifying effect of a proposed treatment, or in early clinical development (e.g., phase 2 studies) to establish proof of concept to support advancement of a potential treatment to a phase 3 trial.

Our results confirm that data‐driven ROIs of change identify expected patterns of atrophy, based on the known patterns of disease in FTLD and the limited prior data on longitudinal change, and improve the reliability of change measurements compared with a priori ROIs and compared with clinical measures. The method is most beneficial in situations where regions of maximal change are least variable across individuals. Future studies can try to improve the reliability of tracking change in bvFTD by attempting to identify factors that predict the regions most likely to change. The approach used here is one of many data‐driven methods used to optimize voxel‐wise analyses in both cross‐sectional and longitudinal studies (Avants et al., 2010; Chen et al., 2010; Hua et al., 2009; McMillan et al., 2014; Reddan, Lindquist, & Wager, 2017; Vounou et al., 2012). Our method is similar to prior studies that used training and test sets (Chen et al., 2010; Hua et al., 2009) but instead of a single training‐test partition, we use cross‐validation through repeated resampling of the data. The estimate of the effect size we generated from this procedure should be a conservative estimate of the effect size achievable with the optimized ROI we created because it was generated based on multiple partitions of the data that always used a smaller sample than the total (the training partition) to generate a ROI that was then tested in the test partition for each cross‐validation run. It will be important to test the effect size of the final consensus ROI in independent datasets from different cohorts, ideally collected at other centers. In addition, our analysis compared the rates of change in cerebral cortex to change in a limited number of cognitive and functional variables. Future studies should compare rates of change in imaging with a larger number of clinical variables to better evaluate the relative utility of imaging. Lastly, while all the cases analyzed were recruited for longitudinal follow‐up, the studies did not employ true clinical trial methodology with systematic follow‐up of every case. Thus, clinical features that influence enrollment criteria or dropout might differ in clinical trials and thus be associated with different effect sizes for longitudinal change.

## Conflict of Interest

None declared.

## References

[brb3675-bib-0001] Ard, M. C. , Raghavan, N. , & Edland, S. D. (2015). Optimal composite scores for longitudinal clinical trials under the linear mixed effects model. Pharmaceutical Statistics, 14, 418–426. 26223663 10.1002/pst.1701 PMC5132034

[brb3675-bib-0002] Ashburner, J. (2007). A fast diffeomorphic image registration algorithm. NeuroImage, 38, 95–113. 17761438 10.1016/j.neuroimage.2007.07.007

[brb3675-bib-0003] Ashburner, J. , & Ridgway, G. R. (2012). Symmetric diffeomorphic modeling of longitudinal structural MRI. Frontiers in Neuroscience, 6, 197. 23386806 10.3389/fnins.2012.00197 PMC3564017

[brb3675-bib-0004] Avants, B. B. , Cook, P. A. , Ungar, L. , Gee, J. C. , & Grossman, M. (2010). Dementia induces correlated reductions in white matter integrity and cortical thickness: A multivariate neuroimaging study with sparse canonical correlation analysis. NeuroImage, 50, 1004–1016. 20083207 10.1016/j.neuroimage.2010.01.041 PMC2953719

[brb3675-bib-0005] Bang, J. , Spina, S. , & Miller, B. L. (2015). Frontotemporal dementia. The Lancet, 386, 1672–1682. 10.1016/S0140-6736(15)00461-4 PMC5970949 26595641

[brb3675-bib-0006] Birn, R. M. , Kenworthy, L. , Case, L. , Caravella, R. , Jones, T. B. , Bandettini, P. A. , & Martin, A. (2010). Neural systems supporting lexical search guided by letter and semantic category cues: A self‐paced overt response fMRI study of verbal fluency. NeuroImage, 49, 1099–1107. 19632335 10.1016/j.neuroimage.2009.07.036 PMC2832834

[brb3675-bib-0007] Boxer, A. L. , & Boeve, B. F. (2007). Frontotemporal dementia treatment: Current symptomatic therapies and implications of recent genetic, biochemical, and neuroimaging studies. Alzheimer Disease and Associated Disorders, 21, S79–S87. 18090429 10.1097/WAD.0b013e31815c345e

[brb3675-bib-0008] Boxer, A. L. , Gold, M. , Huey, E. , Hu, W. T. , Rosen, H. , Kramer, J. , … Cummings, J. L. (2013). The advantages of frontotemporal degeneration drug development (part 2 of frontotemporal degeneration: The next therapeutic frontier). Alzheimer's & Dementia: The Journal of the Alzheimer's Association, 9, 189–198. 10.1016/j.jalz.2012.03.003 PMC3562382 23062850

[brb3675-bib-0009] Boxer, A. L. , Knopman, D. S. , Kaufer, D. I. , Grossman, M. , Onyike, C. , Graf‐Radford, N. , … Miller, B. L. (2013). Memantine in patients with frontotemporal lobar degeneration: A multicentre, randomised, double‐blind, placebo‐controlled trial. The Lancet Neurology, 12, 149–156. 23290598 10.1016/S1474-4422(12)70320-4 PMC3756890

[brb3675-bib-0010] Brambati, S. M. , Rankin, K. P. , Narvid, J. , Seeley, W. W. , Dean, D. , Rosen, H. J. , … Gorno‐Tempini, M. L. (2009). Atrophy progression in semantic dementia with asymmetric temporal involvement: A tensor‐based morphometry study. Neurobiology of Aging, 30, 103–111. 17604879 10.1016/j.neurobiolaging.2007.05.014 PMC2643844

[brb3675-bib-0011] Chen, K. , Langbaum, J. B. , Fleisher, A. S. , Ayutyanont, N. , Reschke, C. , Lee, W. , … Reiman, E. M. (2010). Twelve‐month metabolic declines in probable Alzheimer's disease and amnestic mild cognitive impairment assessed using an empirically pre‐defined statistical region‐of‐interest: Findings from the Alzheimer's Disease Neuroimaging Initiative. NeuroImage, 51, 654–664. 20202480 10.1016/j.neuroimage.2010.02.064 PMC2856742

[brb3675-bib-0012] Delis, D. , Kaplan, E. B. , & Kramer, J. (2001). The Delis‐Kaplan executive function system. San Antonio, TX: The Psychological Corporation.

[brb3675-bib-0013] Delis, D. C. , Kramer, J. H. , Kaplan, E. , & Ober, B. A. (2000). California verbal learning test (2nd ed.). San Antonio, TX: The Psychological Corporation.

[brb3675-bib-0014] Dickerson, B. C. , Stoub, T. R. , Shah, R. C. , Sperling, R. A. , Killiany, R. J. , Albert, M. S. , … Detoledo‐Morrell, L. (2011). Alzheimer‐signature MRI biomarker predicts AD dementia in cognitively normal adults. Neurology, 76, 1395–1402. 21490323 10.1212/WNL.0b013e3182166e96 PMC3087406

[brb3675-bib-0015] Folstein, M. F. , Folstein, S. E. , & McHugh, P. R. (1975). “Mini‐mental state”. A practical method for grading the mental state of patients for the clinician. Journal of Psychiatric Research, 12, 189–198. 1202204 10.1016/0022-3956(75)90026-6

[brb3675-bib-0016] Friston, K. J. , Holmes, A. , Poline, J. B. , Price, C. J. , & Frith, C. D. (1996). Detecting activations in PET and fMRI: Levels of inference and power. NeuroImage, 4, 223–235. 9345513 10.1006/nimg.1996.0074

[brb3675-bib-0017] Gordon, E. , Rohrer, J. D. , Kim, L. G. , Omar, R. , Rossor, M. N. , Fox, N. C. , & Warren, J. D. (2010). Measuring disease progression in frontotemporal lobar degeneration: A clinical and MRI study. Neurology, 74, 666–673. 20177120 10.1212/WNL.0b013e3181d1a879 PMC2830919

[brb3675-bib-0018] Gorno‐Tempini, M. L. , Hillis, A. E. , Weintraub, S. , Kertesz, A. , Mendez, M. , Cappa, S. F. , … Grossman, M. (2011). Classification of primary progressive aphasia and its variants. Neurology, 76, 1006–1014. 21325651 10.1212/WNL.0b013e31821103e6 PMC3059138

[brb3675-bib-0019] Hua, X. , Lee, S. , Yanovsky, I. , Leow, A. D. , Chou, Y. Y. , Ho, A. J. , … Thompson, P. M. (2009). Optimizing power to track brain degeneration in Alzheimer's disease and mild cognitive impairment with tensor‐based morphometry: An ADNI study of 515 subjects. NeuroImage, 48, 668–681. 19615450 10.1016/j.neuroimage.2009.07.011 PMC2971697

[brb3675-bib-0020] Jack Jr, C. R. , Knopman, D. S. , Jagust, W. J. , Petersen, R. C. , Weiner, M. W. , Aisen, P. S. , … Trojanowski, J. Q. (2013). Tracking pathophysiological processes in Alzheimer's disease: An updated hypothetical model of dynamic biomarkers. The Lancet Neurology, 12, 207–216. 23332364 10.1016/S1474-4422(12)70291-0 PMC3622225

[brb3675-bib-0021] Jack Jr, C. R. , Petersen, R. C. , Xu, Y. C. , Waring, S. C. , O'Brien, P. C. , Tangalos, E. G. , … Kokmen, E. (1997). Medial temporal atrophy on MRI in normal aging and very mild Alzheimer's disease. Neurology, 49, 786–794. 9305341 10.1212/wnl.49.3.786 PMC2730601

[brb3675-bib-0022] Kaplan, E. , Goodglass, H. , & Wintraub, S. (1983). The Boston naming test. Philadelphia, PA: Lea and Febiger.

[brb3675-bib-0023] Knopman, D. S. , Jack Jr, C. R. , Kramer, J. H. , Boeve, B. F. , Caselli, R. J. , Graff‐Radford, N. R. , … Mercaldo, N. D. (2009). Brain and ventricular volumetric changes in frontotemporal lobar degeneration over 1 year. Neurology, 72, 1843–1849. 19470967 10.1212/WNL.0b013e3181a71236 PMC2690986

[brb3675-bib-0024] Knopman, D. S. , Kramer, J. H. , Boeve, B. F. , Caselli, R. J. , Graff‐Radford, N. R. , Mendez, M. F. , … Mercaldo, N. (2008). Development of methodology for conducting clinical trials in frontotemporal lobar degeneration. Brain, 131, 2957–2968. 18829698 10.1093/brain/awn234 PMC2725027

[brb3675-bib-0025] Kramer, J. H. , Jurik, J. , Sha, S. J. , Rankin, K. P. , Rosen, H. J. , Johnson, J. K. , & Miller, B. L. (2003). Distinctive neuropsychological patterns in frontotemporal dementia, semantic dementia, and Alzheimer disease. Cognitive and Behavioral Neurology, 16, 211–218. 14665820 10.1097/00146965-200312000-00002

[brb3675-bib-0026] Kril, J. J. , & Halliday, G. M. (2004). Clinicopathological staging of frontotemporal dementia severity: Correlation with regional atrophy. Dementia and Geriatric Cognitive Disorders, 17, 311–315. 15178943 10.1159/000077161

[brb3675-bib-0027] Krueger, C. E. , Dean, D. L. , Rosen, H. J. , Halabi, C. , Weiner, M. , Miller, B. L. , & Kramer, J. H. (2010). Longitudinal rates of lobar atrophy in frontotemporal dementia, semantic dementia, and Alzheimer's disease. Alzheimer Disease and Associated Disorders, 24, 43–48. 19571735 10.1097/WAD.0b013e3181a6f101 PMC2837112

[brb3675-bib-0028] Mahoney, C. J. , Simpson, I. J. , Nicholas, J. M. , Fletcher, P. D. , Downey, L. E. , Golden, H. L. , … Fox, N. C. (2015). Longitudinal diffusion tensor imaging in frontotemporal dementia. Annals of Neurology, 77, 33–46. 25363208 10.1002/ana.24296 PMC4305215

[brb3675-bib-0029] Maldjian, J. A. , Laurienti, P. J. , Kraft, R. A. , & Burdette, J. H. (2003). An automated method for neuroanatomic and cytoarchitectonic atlas‐based interrogation of fMRI data sets. NeuroImage, 19, 1233–1239. 12880848 10.1016/s1053-8119(03)00169-1

[brb3675-bib-0030] McKhann, G. , Drachman, D. , Folstein, M. , Katzman, R. , Price, D. , & Stadlan, E. M. (1984). Clinical diagnosis of Alzheimer's disease: Report of the NINCDS‐ADRDA Work Group under the auspices of Department of Health and Human Services Task Force on Alzheimer's Disease. Neurology, 34, 939–944. 6610841 10.1212/wnl.34.7.939

[brb3675-bib-0031] McMillan, C. T. , Avants, B. B. , Cook, P. , Ungar, L. , Trojanowski, J. Q. , & Grossman, M. (2014). The power of neuroimaging biomarkers for screening frontotemporal dementia. Human Brain Mapping, 35, 4827–4840. 24687814 10.1002/hbm.22515 PMC4107021

[brb3675-bib-0032] Morris, J. C. (1997). Clinical dementia rating: A reliable and valid diagnostic and staging measure for dementia of the Alzheimer type. International Psychogeriatrics, 9, 173–176; discussion 177–178. 9447441 10.1017/s1041610297004870

[brb3675-bib-0033] Naasan, G. , Rabinovici, G. D. , Ghosh, P. , Elofson, J. D. , Miller, B. L. , Coppola, G. , … Rosen, H. J. (2016). Amyloid in dementia associated with familial FTLD: Not an innocent bystander. Neurocase, 22, 76–83. 26040468 10.1080/13554794.2015.1046458 PMC4662906

[brb3675-bib-0034] Neary, D. , Snowden, J. S. , Gustafson, L. , Passant, U. , Stuss, D. , Black, S. , … Benson, D. F. (1998). Frontotemporal lobar degeneration: A consensus on clinical diagnostic criteria. Neurology, 51, 1546–1554. 9855500 10.1212/wnl.51.6.1546

[brb3675-bib-0035] Pankov, A. , Binney, R. J. , Staffaroni, A. M. , Kornak, J. , Attygalle, S. , Schuff, N. , … Rosen, H. J. (2016). Data‐driven regions of interest for longitudinal change in frontotemporal lobar degeneration. NeuroImage Clinical, 12, 332–340. 27547726 10.1016/j.nicl.2015.08.002 PMC4983147

[brb3675-bib-0036] Papageorgiou, S. G. , Kontaxis, T. , Bonakis, A. , Kalfakis, N. , & Vassilopoulos, D. (2009). Frequency and causes of early‐onset dementia in a tertiary referral center in Athens. Alzheimer Disease and Associated Disorders, 23, 347–351. 19568157 10.1097/WAD.0b013e31819e6b28

[brb3675-bib-0037] Rascovsky, K. , Hodges, J. R. , Knopman, D. , Mendez, M. F. , Kramer, J. H. , Neuhaus, J. , … Miller, B. L. (2011). Sensitivity of revised diagnostic criteria for the behavioural variant of frontotemporal dementia. Brain, 134, 2456–2477. 21810890 10.1093/brain/awr179 PMC3170532

[brb3675-bib-0038] Rascovsky, K. , Salmon, D. P. , Lipton, A. M. , Leverenz, J. B. , DeCarli, C. , Jagust, W. J. , … Galasko, D. (2005). Rate of progression differs in frontotemporal dementia and Alzheimer disease. Neurology, 65, 397–403. 16087904 10.1212/01.wnl.0000171343.43314.6e

[brb3675-bib-0039] Reddan, M. C. , Lindquist, M. A. , & Wager, T. D. (2017). Effect Size Estimation in Neuroimaging. JAMA Psychiatry, doi: 10.1001/jamapsychiatry.2016.3356. 10.1001/jamapsychiatry.2016.3356 28099973

[brb3675-bib-0040] Roberson, E. D. , Hesse, J. H. , Rose, K. D. , Slama, H. , Johnson, J. K. , Yaffe, K. , … Miller, B. L. (2005). Frontotemporal dementia progresses to death faster than Alzheimer disease. Neurology, 65, 719–725. 16157905 10.1212/01.wnl.0000173837.82820.9f

[brb3675-bib-0041] Rohrer, J. D. , Clarkson, M. J. , Kittus, R. , Rossor, M. N. , Ourselin, S. , Warren, J. D. , & Fox, N. C. (2012). Rates of hemispheric and lobar atrophy in the language variants of frontotemporal lobar degeneration. Journal of Alzheimer's Disease, 30, 407–411. 10.3233/JAD-2012-111556 PMC4606976 22406442

[brb3675-bib-0042] Rohrer, J. D. , McNaught, E. , Foster, J. , Clegg, S. L. , Barnes, J. , Omar, R. , … Fox, N. C. (2008). Tracking progression in frontotemporal lobar degeneration: Serial MRI in semantic dementia. Neurology, 71, 1445–1451. 18955688 10.1212/01.wnl.0000327889.13734.cd

[brb3675-bib-0043] Rosen, H. J. , Gorno‐Tempini, M. L. , Goldman, W. P. , Perry, R. J. , Schuff, N. , Weiner, M. , … Miller, B. L. (2002). Patterns of brain atrophy in frontotemporal dementia and semantic dementia. Neurology, 58, 198–208. 11805245 10.1212/wnl.58.2.198

[brb3675-bib-0044] Schuff, N. , Tosun, D. , Insel, P. S. , Chiang, G. C. , Truran, D. , Aisen, P. S. , … Weiner, M. W. (2012). Nonlinear time course of brain volume loss in cognitively normal and impaired elders. Neurobiology of Aging, 33, 845–855. 20855131 10.1016/j.neurobiolaging.2010.07.012 PMC3032014

[brb3675-bib-0045] Seeley, W. W. , Crawford, R. , Rascovsky, K. , Kramer, J. H. , Weiner, M. , Miller, B. L. , & Gorno‐Tempini, M. L. (2008). Frontal paralimbic network atrophy in very mild behavioral variant frontotemporal dementia. Archives of Neurology, 65, 249–255. 18268196 10.1001/archneurol.2007.38 PMC2544627

[brb3675-bib-0046] Seeley, W. W. , Crawford, R. K. , Zhou, J. , Miller, B. L. , & Greicius, M. D. (2009). Neurodegenerative diseases target large‐scale human brain networks. Neuron, 62, 42–52. 19376066 10.1016/j.neuron.2009.03.024 PMC2691647

[brb3675-bib-0047] Tartaglia, M. C. , Rosen, H. J. , & Miller, B. L. (2011). Neuroimaging in dementia. Neurotherapeutics: The Journal of the American Society for Experimental NeuroTherapeutics, 8, 82–92. 21274688 10.1007/s13311-010-0012-2 PMC3026935

[brb3675-bib-0048] Thomas, A. (2010). The conditional independences between variables derived from two independent identically distributed Markov random fields when pairwise order is ignored. Mathematical Medicine and Biology, 27, 283–288. 19942608 10.1093/imammb/dqp022 PMC2948832

[brb3675-bib-0049] Vounou, M. , Janousova, E. , Wolz, R. , Stein, J. L. , Thompson, P. M. , Rueckert, D. , , … Alzheimer's Disease Neuroimaging Initiative (2012). Sparse reduced‐rank regression detects genetic associations with voxel‐wise longitudinal phenotypes in Alzheimer's disease. NeuroImage, 60, 700–716. 22209813 10.1016/j.neuroimage.2011.12.029 PMC3551466

[brb3675-bib-0050] Weiner, M. W. , Veitch, D. P. , Aisen, P. S. , Beckett, L. A. , Cairns, N. J. , Green, R. C. , … Trojanowski, J. Q. (2013). The Alzheimer's Disease Neuroimaging Initiative: A review of papers published since its inception. Alzheimer's & Dementia: The Journal of the Alzheimer's Association, 9, e111–e194. 10.1016/j.jalz.2013.05.1769 PMC4108198 23932184

[brb3675-bib-0051] Whitwell, J. L. , Jack Jr, C. R. , Parisi, J. E. , Knopman, D. S. , Boeve, B. F. , Petersen, R. C. , … Josephs, K. A. (2011). Imaging signatures of molecular pathology in behavioral variant frontotemporal dementia. Journal of Molecular Neuroscience, 45, 372–378. 21556732 10.1007/s12031-011-9533-3 PMC3401589

[brb3675-bib-0052] Yushkevich, P. A. , Avants, B. B. , Das, S. R. , Pluta, J. , Altinay, M. , & Craige, C. (2010). Bias in estimation of hippocampal atrophy using deformation‐based morphometry arises from asymmetric global normalization: An illustration in ADNI 3 T MRI data. NeuroImage, 50, 434–445. 20005963 10.1016/j.neuroimage.2009.12.007 PMC2823935

